# When tumors speak: Understanding EEG periodic discharges in primary brain tumors and their clinical relevance

**DOI:** 10.1093/nop/npaf106

**Published:** 2025-10-13

**Authors:** Stefano Consoli, Fedele Dono, Giacomo Evangelista, Sara Cipollone, Stefano L Sensi

**Affiliations:** Department of Neuroscience, Imaging and Clinical Science, “G. d’Annunzio” University of Chieti-Pescara (S.C., F.D., G.E., S.C., S.L.S.); Behavioral Neurology and Molecular Neurology Units, Center for Advanced Studies and Technology (CAST) and Institute for Advanced Biomedical Technologies (ITAB), University of Chieti-Pescara, Chieti (S.C., F.D., G.E., S.C., S.L.S.); University “G. d’Annunzio” of Chieti-Pescara (S.C., F.D., G.E., S.C., S.L.S.); Epilepsy Center, Neurology Institute “SS Annunziata” University Hospital, University of Chieti-Pescara, Chieti (S.C., F.D., G.E., S.C., S.L.S.); Department of Neuroscience, Imaging and Clinical Science, “G. d’Annunzio” University of Chieti-Pescara (S.C., F.D., G.E., S.C., S.L.S.); Behavioral Neurology and Molecular Neurology Units, Center for Advanced Studies and Technology (CAST) and Institute for Advanced Biomedical Technologies (ITAB), University of Chieti-Pescara, Chieti (S.C., F.D., G.E., S.C., S.L.S.); University “G. d’Annunzio” of Chieti-Pescara (S.C., F.D., G.E., S.C., S.L.S.); Epilepsy Center, Neurology Institute “SS Annunziata” University Hospital, University of Chieti-Pescara, Chieti (S.C., F.D., G.E., S.C., S.L.S.); Department of Neuroscience, Imaging and Clinical Science, “G. d’Annunzio” University of Chieti-Pescara (S.C., F.D., G.E., S.C., S.L.S.); Behavioral Neurology and Molecular Neurology Units, Center for Advanced Studies and Technology (CAST) and Institute for Advanced Biomedical Technologies (ITAB), University of Chieti-Pescara, Chieti (S.C., F.D., G.E., S.C., S.L.S.); University “G. d’Annunzio” of Chieti-Pescara (S.C., F.D., G.E., S.C., S.L.S.); Epilepsy Center, Neurology Institute “SS Annunziata” University Hospital, University of Chieti-Pescara, Chieti (S.C., F.D., G.E., S.C., S.L.S.); Department of Neuroscience, Imaging and Clinical Science, “G. d’Annunzio” University of Chieti-Pescara (S.C., F.D., G.E., S.C., S.L.S.); Behavioral Neurology and Molecular Neurology Units, Center for Advanced Studies and Technology (CAST) and Institute for Advanced Biomedical Technologies (ITAB), University of Chieti-Pescara, Chieti (S.C., F.D., G.E., S.C., S.L.S.); University “G. d’Annunzio” of Chieti-Pescara (S.C., F.D., G.E., S.C., S.L.S.); Epilepsy Center, Neurology Institute “SS Annunziata” University Hospital, University of Chieti-Pescara, Chieti (S.C., F.D., G.E., S.C., S.L.S.); Department of Neuroscience, Imaging and Clinical Science, “G. d’Annunzio” University of Chieti-Pescara (S.C., F.D., G.E., S.C., S.L.S.); Behavioral Neurology and Molecular Neurology Units, Center for Advanced Studies and Technology (CAST) and Institute for Advanced Biomedical Technologies (ITAB), University of Chieti-Pescara, Chieti (S.C., F.D., G.E., S.C., S.L.S.); University “G. d’Annunzio” of Chieti-Pescara (S.C., F.D., G.E., S.C., S.L.S.); Epilepsy Center, Neurology Institute “SS Annunziata” University Hospital, University of Chieti-Pescara, Chieti (S.C., F.D., G.E., S.C., S.L.S.)

**Keywords:** BTRE, critical decompensation threshold, high-grade glioma, hypoxia, status epilepticus

## Abstract

**Abstract:**

Background Periodic discharges (PDs) are electroencephalogram (EEG) patterns commonly associated with focal cerebral lesions, particularly brain tumors (BTs). The clinical interpretation of PDs and their pathophysiological origins are controversial. This retrospective study describes the prevalence and characteristics of PDs in patients with BT, focusing on their association with BT-related epilepsy (BTRE) and status epilepticus (SE) diagnosis.

**Methods:**

Adult patients with BT who underwent a video-EEG recording were retrospectively selected from the Neurology Institute of “G. d‘Annunzio” University of Chieti-Pescara from January 2016 to January 2024. Demographics, clinical features, tumor characteristics, and radiological findings were collected. Video-EEG data were reviewed to identify PDs.

**Results:**

One hundred and forty patients with BT (mean age: 60.6 ± 17.6 years; 61 females) were enrolled. Fifteen patients (10.7%) exhibited PDs on video-EEG (PD+ group). In the PD+ group, lateralized PDs (LPDs) were most observed (93.3%). Periodic discharges were concordant with the side of the tumor lesion in 73.3% of cases. Compared to the PD− group, patients in the PD+ group more frequently showed a parietal (*P* = .0019) and occipital BT localization (*P* < .0001), a high-grade tumor histology (*P* = .048) and a previous BTRE diagnosis (*P* = .014). SE was observed in 4 patients (26.7%), in association with a significantly higher PDs frequency (SE patients: 1.6 Hz, non-SE patients: 0.8 Hz; *P* = .003).

**Conclusions:**

PDs are more commonly observed in patients with high-grade primary BT, particularly those with parietal or occipital localization. They are predominantly reported in individuals with a prior diagnosis of BTRE and may indicate SE, especially when their frequency exceeds 1.6 Hz.

Key points

- About 10% of brain tumor (BT) patients show periodic discharges (PDs).
- PDs occur mostly in high-grade gliomas with a parieto-occipital localization.
- In our cohort, PDs > 1.6 Hz in high-grade tumors were associated SE.

Importance of the Study

Our findings reveal that approximately 10% of BT patients exhibit Periodic Discharges (PDs), with significantly higher prevalence in individuals with high-grade BT, particularly those with parietal or occipital localization. Periodic discharges likely result from dysregulation of feed-forward inhibitory circuits and disruptions in thalamocortical homeostasis. Patients with a history of BT-related epilepsy (BTRE) are at an elevated risk of developing PDs, likely due to preexisting cortical excitability changes. Furthermore, in high-grade primary BT, PDs exceeding a frequency of 1.6 Hz are strongly correlated with the occurrence of status epilepticus (SE), suggesting that PDs may serve as an important marker of this condition. These findings indicate that PDs may act as a biomarker for SE in patient with primary BT, reflecting specific tumor localizations, grades, and prior epileptic conditions.

Brain tumors (BTs) are one of the top 10 causes of cancer-related death worldwide.^1^^,^^2^ Classically, they are divided into 2 subgroups: primary (ie, neoplasms that arose from neurons, astrocytes, or meningeal cells) and secondary (ie, metastasis derived from systemic solid or hematological malignancies).^3^ According to the World Health Organization (WHO) classification, primary BT can be divided into low and high grades according to their histological and molecular features.^3–5^

Seizures are 1 of the most common symptoms of primary BT; the onset can be observed at the diagnosis and during the disease course.^6^ Thus, BT-related epilepsy (BTRE) is 1 of the most frequently observed comorbidities in patients suffering from BT, with an incidence ranging from 30% to 100% depending on the tumor type.^6^^,^^7^ Slow-growing tumors (eg, neuroepithelial dysembryoplastic tumors and gangliogliomas) are frequently epileptogenic and associated with a higher risk of seizures, contrarywise to high-grade gliomas, which have a relatively lower one.^6–8^

The electroencephalogram (EEG) is a key neurophysiological technique in supporting the diagnosis and guiding the therapeutic management of patients with BTRE.^9^ Several EEG abnormalities may be encountered, including focal/diffuse slow-wave activity in theta or delta range that increases during the hyperventilation and epileptiform discharges (ie, spike, spike-wave and sharp-wave) both in the tumor and peri-tumoral areas.^10^ Up to 10% of patients with a primary BT may show Periodic Discharges (PDs),^10^^,^^11^ which refers to an EEG pattern characterized by a waveform that recurs at almost regular intervals with uniform morphology and duration and is spaced by a regular and discernible inter-discharge period.^12^ Based on the EEG localization, PDs are defined as generalized (GPDs) or lateralized (LPDs).^12^ Almost half of severely ill patients may present PDs during continuous EEG (cEEG) monitoring.^13^^,^^14^ While GPDs typically signify diffuse brain ­dysfunction commonly observed in conditions such as anoxic, toxic-metabolic, or infectious diseases, LPDs are linked to focal cerebral injuries, primarily cerebrovascular events (eg, ischemic stroke, intracerebral hemorrhage, and subdural hematoma), or structural brain lesions (eg, abscesses and BTs).^15^^,^^16^ PDs are not consistently regarded as an ictal pattern, often situated within an interictal-ictal continuum (IIC). Indeed, according to the 2021 American Clinical Neurophysiology Society (ACNS) guidelines,^12^ various PD characteristics (eg, frequency, morphology, localization, and fluctuation) may aid in differentiating PDs strongly associated with ictal activity [eg, status epilepticus (SE)] from those merely indicating an underlying brain structural abnormality.^12^^,^^17^

This study aims to describe the prevalence and characteristics of PDs in patients with BT, focusing on their association with BTRE and SE diagnosis.

## Methods

### Patients’ Selection and Evaluation

Adult patients with primary BT were retrospectively selected from the database of the Neurology Clinic of “G. d’Annunzio” University of Chieti-Pescara from January 2016 to January 2024. The following inclusion criteria were employed: (1) adult subjects aged over 18 years; (2) patients diagnosed with BT assessed according to WHO criteria and possibly undergoing standard antitumor therapies [chemotherapy (CT), radiotherapy (RT), and/or total or partial surgical interventions on BT]; (3) patients underwent at least one standard 21-channel video-EEG (vEEG) according to the routine clinical practice.

The EEGs were obtained as part of routine clinical care in patients presenting to the Emergency Department with new-onset seizures, changes in seizure semiology, altered mental status, or unexplained neurological decline. Most of the EEGs were performed in the outpatient setting during scheduled follow-up visits, typically once every 3-6 months per year, in the context of ongoing seizure monitoring or neurological evaluation. Only a minority of recordings were conducted during hospitalizations. No continuous or ICU EEG monitoring was included. The average EEG recording duration was 20-30 min using the standard 10-20 systems montage.

Demographic data, clinical features, tumor characteristics (including histology, localization, lateralization, grade, and size), treatment details, and radiological findings were collected. The diagnosis of BTRE and SE was reassessed according to the criteria established by the International League Against Epilepsy (ILAE). The BT subtypes were identified in accordance with the 2021 WHO Classification of Tumors of the Central Nervous System. Consequently, whenever possible, all BT cases diagnosed before 2021 were reviewed and reclassified according to these updated criteria. If this was not possible, we maintained the previously reported classification.

Two groups of expert neurophysiologists (group 1: F.D. and S.C.; group 2: G.E. and S.L.S.) analyzed video-EEG data to identify patients exhibiting PDs. PDs were described according to the ACNS Standardized Critical Care EEG Terminology. An inter-group comparison was performed to identify any inter-rater reliability and consistency in identifying PDs.

Accordingly, patients were categorized into PD+ and PD− groups based on the presence of a PD pattern. Demographic, clinical, radiological, and neurophysiological data were then compared between the two groups. The study received approval from the local ethics committee and complied with the Declaration of Helsinki.

### Statistical Analysis

Statistical analysis was performed on the final dataset containing all information pooled from the patients’ data. Data were reported as the median plus interquartile range (IQR) or absolute number and percentage for continuous or categorical and dichotomous variables, respectively. Data were analyzed in Statistical Package for Social Science (SPSS^®^) software version 22 (SPSS, Inc.). The normality of continuous data was checked via the Kolmogorov-Smirnov test. Differences in the frequency of clinical manifestations between the 2 groups were assessed with the χ^2^ test. The alpha level was set at 0.05 for statistical significance. Differences between patient groups were tested using the Mann-Whitney test or the Chi-square test, as appropriate.

## Results

### Demographics and BT Characteristics

One hundred forty patients (61 females, 43.6%) were enrolled. The mean age at diagnosis was 60.6 ± 17.6 years. According to the 2021 WHO Classification of Tumors of the Central Nervous System,^3^ 105 patients had a diffuse glioma: particularly, 80 patients had isocitrate dehydrogenase (IDH) wild-type glioblastomas, 17 IDH-mutant astrocytomas, 8 oligodendrogliomas (IDH-mutant and 1p19q co-deletion), 32 meningiomas, and 3 had primary CNS lymphoma (PCNSL). Most patients exhibited high-grade (WHO 3-4) tumors (100/140, 71.4%). Among the 17 IDH-mutant astrocytomas, 6 were WHO Grade 2, 7 were WHO Grade 3, and 4 were WHO Grade 4. Of the 8 patients with oligodendrogliomas, 7 were WHO Grade 2 and 1 was WHO Grade 3. Additionally, among the 32 meningiomas, 22 were classified as low-grade (WHO Grade 1) and 10 as high-grade (WHO Grade 2 or 3). Half of the patients had a frontal BT (72/140, 51.4%), followed by temporal lobe (45/140, 32.1%), parietal (44/140, 31.4%), and occipital lobe (13/140, 9.3%), localization. Sixty-one patients had a multi-lobar BT (61/140, 43.6%). Right and left lateralization were 51 (36.4%) and 76 (54.3%) cases, respectively, with involvement of both hemispheres in 13 (9.3%) patients.

Additional demographic data are reported in Table 1.

**Table 1.** Demographic data

	Total patients (*n* = 140)	PD+ GROUP (*n* = 15)	PD− GROUP (*n* = 125)	*P*-value
Age at diagnosis (years)	60.6 ± 17.6	68.7 ± 11.4	59.6 ± 18	.032
Sex				
• Male	79 (56.4%)	12	67	.014
• Female	61 (43.6%)	3	58	.014
Primary brain tumor				
• Gliomas	105 (75%)	14	91	.08
- Glioblastoma	80 (57.1%)	12	68	.058
- Astrocytoma	17 (12.1%)	2	15	.88
- Oligodendroglioma	8 (5.7%)	0	8	-
• Meningioma	32 (22.9%)	1	31	.11
• Lymphoma	3 (2.1%)	0	3	-
Tumor stage				
• Low grade (1-2)	40 (28.6%)	1	39	.048
• High grade (3-4)	100 (71.4%)	14	86	.048
Tumor localization				
• Frontal lobe	72 (51.4%)	5	67	.14
• Temporal lobe	45 (32.1%)	4	41	.63
• Parietal lobe	44 (31.4%)	10	34	.0019
• Occipital lobe	13 (9.3%)	7	6	< .00001
• Insular lobe	8 (5.7%)	0	8	-
• Multilobar	61 (43.6%)	10	51	.06
Tumor lateralization				
• Left	76 (54.3%)	7	69	.53
• Right	51 (36.4%)	8	44	.17
• Both	13 (9.3%)	0	13	-
Tumor treatment				
• Surgery	70 (50%)	7	63	.79
• Radiotherapy	41 (29.3%)	6	35	.33
• Chemotherapy	40 (28.6%)	7	33	.1

### Epilepsy and Electroencephalographic Features

Half of the patients (70/140, 50%) had a diagnosis of BTRE, mostly diagnosed at BT disease onset (43/70, 61.4%). Forty-two (40/70, 60%) patients presented only focal seizures, 22 (22/70, 31.4%) focal and focal-to-bilateral tonic-clonic seizures, whereas 6 (8.6%) primary generalized ones.

One hundred and six (106/140, 75.7%) patients were treated with antiseizure medications (ASMs) either after having received a diagnosis of BTRE or as a prophylaxis therapy (36/140, 25.7%). The most used ASM was levetiracetam (LEV), administered in 93 (87.7%) patients, followed by lacosamide (LCS) in 14 (13.2%) patients. Most patients received a single ASM (80/106, 75.5%), while 26 (26/106, 24.5%) were treated with 2 or more (mean ASM number: 1.3 ± 0.6).

Patients underwent a median of 2.1 ± 1.5 electroencephalographic (EEG) recordings. According to the tumor localization, most EEGs (106/140, 75.7%) showed focal slow-wave activity in theta-delta bands. Nineteen patients (19/140, 13.6%) showed focal interictal epileptiform discharges, whereas PDs were recorded in 15 (10.7%) (ie, 14 with LPD and 1 with GPD). The inter-rater reliability and consistency in identifying PDs were 100%.

Additional epilepsy features, treatment details, and EEG data are reported in Table 2.

**Table 2.** Epilepsy features, treatment details, and EEG data

	Total patients (*n* = 140)	PD+ group (*n* = 15)	PD− group (*n* = 125)	*P*-value
Previous epilepsy diagnosis				
• Yes	70 (50%)	12	58	.014
- Focal	42	9	33	
- Focal to bilateral	22	3	19	
- Tonic-clonic	6	0	6	
Antiseizures medication (ASMs)				
• Average number	1.3 ± 0.6	1.4 ± 0.9	1.1 ± 0.9	.28
- Levetiracetam	93	14	77	
- Lacosamide	14	4	10	
- Perampanel	10	1	9	
- Lamotrigine	5	0	5	
- Valproate	2	0	2	
EEG features				
• Focal slow waves	106 (75.7%)	0	106	-
• Interictal ED	19 (13.6%)	0	19	-
• Periodic discharges (PDs)	15 (10.7%)	15	0	-
- Lateralized (LPD)	14 (10%)	14	0	-
- Generalized (GPD)	1 (0.7%)	1	0	-
• No. EEG per patient	2.1 ± 1.5	2.5 ± 1.4	2.5 ± 2.1	.93
• Follow-up (months)	11 ± 21	6.9 ± 11.8	12.4 ± 20.9	.32

ED = epileptiform discharges.

### Periodic Pattern and Associated Clinical Findings

**Figure 1.**
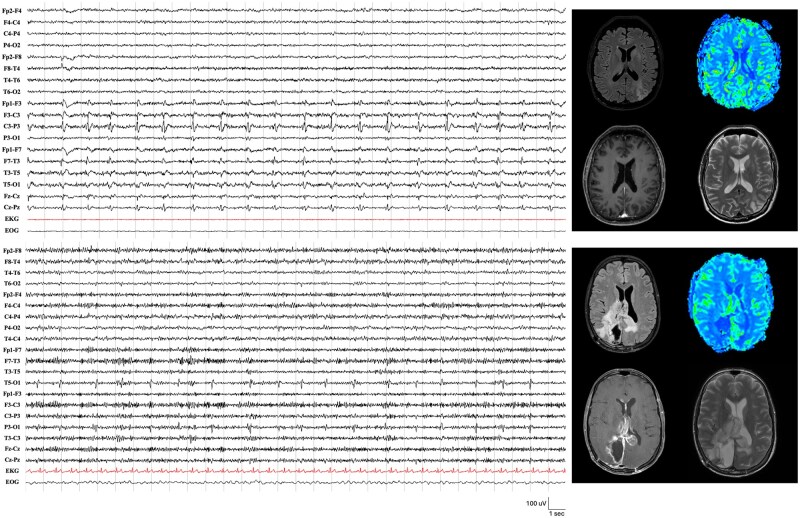
Brain MRI and EEG demonstrating periodic discharges (PD) in 2 patients with brain tumors. (A) EEG reveals lateralized periodic discharges (LPDs) predominantly over the left centro-parietal region, occurring at a frequency of 1 Hz. Corresponding brain MRI shows an irregular, space-occupying lesion in the left parietal lobe, characterized by hypointense signals on T1-weighted images and hyperintense signals on T2/FLAIR sequences. The lesion exerts a mass effect and is surrounded by vasogenic edema. MR perfusion imaging demonstrates elevated relative cerebral blood volume (rCBV) in the affected region. (B) EEG shows LPDs over the left posterior temporal and occipital region, occurring at a frequency of 0.5 Hz. Brain MRI reveals a large, heterogeneous mass involving the right temporoparieto-occipital lobes, extending across the splenium of the corpus callosum into the left parieto-occipital lobes. MRI perfusion imaging highlights elevated rCBV predominantly in the left parieto-occipital region. Abbreviation: EEG, electroencephalogram; FLAIR, fluid-attenuated inversion recovery; MRI, magnetic resonance imaging.

Fifteen patients (12 males, 68.7 ± 11.4 years) had a periodic pattern on EEG. Fourteen patients (14/15, 93.3%) had a high-grade glioma, while 1 (1/15, 6.7%) had a WHO Grade 2 meningioma. According to tumor localization, 10 patients showed a parietal BT (10/15, 66.7%), 7 occipital (7/15, 46.7%), 4 frontal (4/15, 26.7%), and 4 temporal (4/15, 26.7%) localization. Figure 1 illustrates 2 examples of patients with a PD pattern correlated with brain MRI findings.

A diagnosis of BTRE was reported in 12 cases (12/15, 80%), characterized by focal seizures in all cases, with focal-to-bilateral seizures in 3 (3/15, 20%) patients. The mean PD frequency was 1.1 ± 0.5 Hz. PDs were localized according to the tumor site in 11 of 15 cases (73.3%), occurring on average 3.8 months after BT diagnosis, or concurrently with tumor recurrence following surgical and adjuvant therapies. In 4 cases (26.7%) PDs were detected concomitantly with a *de novo* high-grade BT diagnosis.

### Group Comparison

Compared to the PD− group, patients in the PD+ group more often presented a parietal (*P* = .0019) and occipital (*P* < .00001) lesion localization with high-grade histology (*P* = .048). A statistically significant difference was observed in both age at diagnosis (*P* = .032) and sex distribution (*P* = .014) between patients with and without PDs. Specifically, patients in the PD+ group were diagnosed at a slightly older age (68.7 vs 59.6), and female sex was less frequently associated with the presence of PDs. No differences were observed in terms of BT subtype, lateralization, or tumor treatment. Furthermore, no differences between the EEG number (*P* = .93) and follow-up duration (*P* = .32) were observed between the two groups.

Among the PD+ group, 5 patients (4 men, age: 65.6 ± 9.2 years) (5/15, 33.3%) were presented with SE. Three patients (3/5, 60%) had a previous diagnosis of BTRE successfully treated with LEV. According to BT localization, 1 patient had a frontal lobe localization, 2 had a parieto-occipital, and 1 had a temporal, whereas 1 patient showed a selective involvement of the splenium of the corpus callosum. Several SE clinical features were observed, including 2 patients with a convulsive focal motor SE, 2 with aphasic SE, and 1 with a nonconvulsive SE (NCSE) without coma. SE was treated with the administration of benzodiazepine (ie, intravenous diazepam 10-20 mg or lorazepam 4-8 mg) followed by a single or combined second-line ASM therapy (eg, sodic phenytoin 15-18 mg/kg, phenobarbital 15-20 mg/kg, LEV 30-40 mg/kg, valproate 20-30 mg/kg, LCS 200-600 mg, topiramate 20 mg/kg, perampanel 12-20 mg, and brivaracetam 100-200 mg). A higher mean PD frequency was noted in patients diagnosed with SE (SE: 1.6 ± 0.7 Hz; No-SE: 0.8 ± 0.2 Hz) (*P* = .0037). Furthermore, when comparing patients diagnosed with SE to those without, no significant differences were observed in terms of age (*P* = .13), sex (*P* = 1), tumor type (*P* = .17), localization (ie, frontal *P* = .68, parietal *P* = .12, temporal *P* = .44, occipital *P* = .16), or EEG characteristics (ie, plus modifiers *P* = 1).

Additional data are reported in Table 3.

**Table 3.** PD+ group with and without status epilepticus (SE) findings

	PD with SE (*n* = 5)	PD without SE (*n* = 10)	*P*-value
Age	65.6 ± 9.2	74.8 ± 10.7	.13
Sex			1
• M	4	8	
• F	1	2	
Previous BTRE diagnosis			.69
• Yes	3	7	
• No	2	3	
Primary brain tumor			
• Gliomas	5	9	-
- Glioblastoma	3	9	.17
- Astrocytoma	2	0	-
• Meningioma	0	1	-
Tumor stage			
• Low grade (1-2)	0	1	-
• High grade (3-4)	5	9	-
BT localization			
• Frontal	1	3	.68
• Parietal	2	8	.12
• Temporal	1	3	.44
• Occipital	2	5	.16
• Corpus callosum	1	0	-
EEG features			
• Periodic discharges (PDs)			
- Lateralized (LPD)	4	10	-
- Generalized (GPD)	1	0	-
• Frequency (Hz)	1.6 ± 0.7	0.8 ± 0.2	.0037
• Plus modifiers	1	2	1

## Discussion

**Figure 2.**
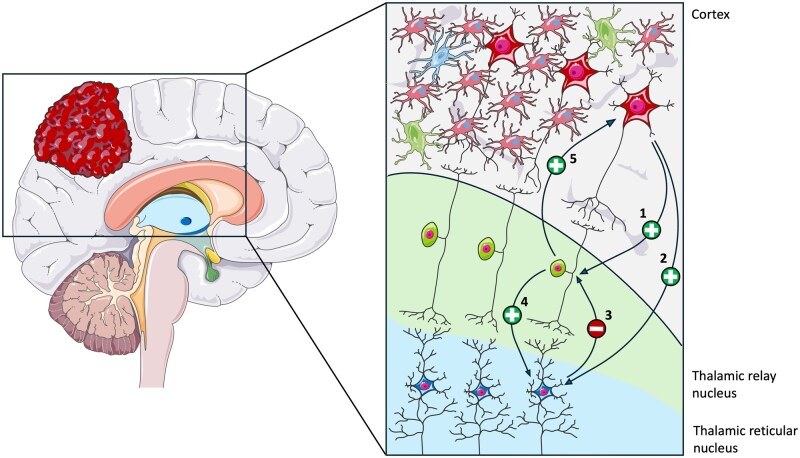
The thalamocortical model. Excitatory neurons in the cortical layers project to both (1) excitatory neurons in the thalamic relay nucleus (SRN) and (2) inhibitory interneurons in the thalamic reticular nuclei (TRN) through a feed-forward inhibitory pathway. Upon stimulation, the TRN neurons inhibit (3) the SRN neurons, thereby reducing excitatory outflow toward the cortex. Additionally, excitatory neurons in the SRN project to both inhibitory neurons in the TRN (4) and excitatory neurons in the cortex (5), thereby regulating the timing of pyramidal cell firing. In patients with brain tumors (BTs), disruption of this circuitry leads to the generation of periodic discharges (PDs).

Although low-grade gliomas are typically associated with higher epileptogenic potential due to their slow growth and cortical involvement, our study highlights that PDs are mainly observed in patients with primary high-grade BT.^18^ This likely reflects distinct pathophysiological mechanisms, whereby high-grade lesions may induce a more profound disruption of thalamocortical circuits (TCC), facilitating the emergence of periodic EEG patterns.^19^ Thus, epileptogenicity and the occurrence of LPDs may represent independent, though not mutually exclusive, phenomena. However, the pathophysiological mechanisms driving PD development in patients with high-grade BT remain incompletely understood.^18^ Experimental findings indicate that PDs arise primarily from synaptic dysfunction, largely driven by neurotransmitter imbalances and ischemic or metabolic neuronal injury,^20^ processes that can disrupt thalamocortical circuit (TCC) homeostasis.^21^ The TCC is crucial for generating brain rhythms, a process mediated through the thalamic reticular nucleus (TRN) and its interactions with the thalamic nuclei and cortical regions.^19^^,^^21^ Disturbances within this network lead to significant network dysregulation often resulting from impaired GABAergic signaling and excessive glutamatergic activity.^22^^,^^23^ A recent computational model has further elucidated TCC dysfunction dynamics in PD generation^24^ (Figure 2). According to this model, cortical pyramidal cells, via glutamatergic pathways, can initiate homosynaptic (excitatory pyramidal cells acting on other pyramidal neurons) and heterosynaptic (excitatory pyramidal cells acting on GABAergic interneurons in the TRN) excitation. The processes activate an inhibitory feedback loop driven by GABAergic transmission.^24^^,^^25^ Under physiological conditions, this “feed-forward inhibitory network” generates alpha-band rhythms; however, alteration of the synaptic glutamatergic pathway from pyramidal cells to interneurons may induce PDs.^25^ Additionally, the model emphasizes the critical role of GABAergic neuron deficits in generating PDs, highlighting their specific contribution to maintaining TCC homeostasis. Due to their high oxygen demand, GABAergic neurons are particularly susceptible to hypoxia,^26^ which is often present in high-grade BT patients as a result of the tumor and peri-tumoral tissues’ tendency toward hypoxic conditions.^27^ Moreover, high-grade BT exhibits a distinctive metabolic adaptation known as the Warburg effect,^28^^,^^29^ in which cancer cells predominantly utilize aerobic glycolysis for energy production despite sufficient oxygen availability.^30^ This metabolic reprogramming increases glycolytic rates, elevates lactate levels, and acidifies the tumoral and peri-tumoral environment. Such acidification not only promotes tumor proliferation but may also elevate seizure risk by activating acid-sensing ion channels (ASICs).^31^ Activation of these channels permits sodium ion (Na⁺) influx, inducing membrane depolarization and neuronal excitability.^32^ Above all, this excitatory-inhibitory imbalance is already impaired in patients with BTRE, thus explaining the higher prevalence of PDs in this subgroup of patients.

Nearly two-thirds of our patients with PD presented with parieto-occipital BTs, suggesting a potential variation in cortical susceptibility. It is well established that cortical cytoarchitecture varies by region in alignment with specific functional roles.^33^ The cortex’s 6-layer structure is not uniformly expressed across all areas; for instance, the motor cortex (or agranular cortex) lacks layers II (outer granular) and IV (inner granular) but exhibits prominent development of layers III (inner pyramidal) and V (ganglionic).^33^^,^^34^ Layer V, in particular, contains giant pyramidal cells (Betz cells) with extensive basal dendritic arborization and robust apical dendrites.^35^ This reduced presence of granular layers results in limited exposure of the frontal cortex to stimulation from thalamic relay nuclei. Additionally, studies indicate that Betz cells are less susceptible to hypoxia-induced damage.^36^ In contrast, posterior cortical regions—such as the parietal somatosensory cortex, occipital visual cortex, and primary auditory cortex—are marked by a higher expression of granular layers, stronger thalamocortical connections, and greater hypoxia susceptibility.^37^^,^^38^ Consequently, BTs in posterior cortical regions may disrupt the excitatory/inhibitory balance, leading to alterations in thalamocortical feed-forward mechanisms that ultimately contribute to PDs development.^24^^,^^25^

Notably, a significant difference in sex was observed indicating that the female sex is less associated with the occurrence of PDs. Indeed, substantial differences in brain cytoarchitectural organization and neuronal density between men and women have been documented.^39^^,^^40^ Specifically, evidence highlights that parietal and occipital cortical thickness as well as the density of GABA-A receptors is greater in women than in men.^39^^,^^41–42^ These data have also been confirmed by studies employing magnetic resonance spectroscopy (MRS), which showed higher GABA concentrations in the occipital cortex of women.^39^^,^^43^ These factors collectively suggest that women, due to their distinct structural and neurochemical characteristics, may experience PD less frequently than men.

Finally, the age of the diagnosis was slightly higher in patients with PDs. This result is likely attributable to the older age of onset for high-grade tumors, as generally reported in the literature.^1^

### To Treat or Not to Treat?

According to our data, it is plausible that not all PDs patterns necessarily signify an ictal state, particularly in the presence of discernible structural lesions such as those associated with hypoxic damage, like BT.^13^^,^^17^^,^^44^ Nevertheless, identifying a critical PD threshold frequency at 2.5 Hz has been proposed to more accurately differentiate ictal patterns from those merely categorized as IIC.^12^ This threshold is supported by research indicating that PDs frequencies exceeding 2 Hz are associated with a significant decline in brain tissue oxygenation and cerebral blood flow, possibly contributing to seizure generation.^13^^,^^45^ This implies a critical decompensation threshold beyond which physiological brain autoregulation to adverse external stimuli becomes ineffective.^13^^,^^44^ Although lower-frequency PDs (<2.5 Hz) are not directly considered ictal, they are widely recognized as epileptiform and should not be dismissed, especially in patients with compatible clinical histories. According to the Salzburg Criteria,^46^ any PD frequency may be considered ictal if associated with clinical symptoms or a response to antiseizure treatment. Therefore, treatment decisions should be guided not solely by PD frequency but by the full clinical and electrographic context.

In our cohort, a mean threshold of 1.6 Hz was observed in patients diagnosed with SE and successfully treated, supporting the need for individualized interpretation of PD frequency and avoiding underestimation of potential ictal patterns in the BT population.

### Limitations

We acknowledge several limitations of our study. The relatively small sample size of our cohort, especially within the subgroup of patients presenting with PDs, may reduce the statistical power of our analyses and the reliability of subgroup comparisons. Second, the heterogeneity of tumor types included in our study may introduce variability in the underlying epileptogenic mechanisms. In addition, the population included both patients with and without BTRE, and some patients without epilepsy were receiving antiseizure medications prophylactically according to the clinicians’ choice. This heterogeneity reflects real-world clinical scenarios, but it may introduce additional confounding factors and limit the ability to draw definitive conclusions regarding risk factors associated with the development of PDs. The single-center design of our study may reflect institution-specific protocols for EEG acquisition, interpretation, and clinical decision-making. This could influence the detection, classification, and reported prevalence of EEG patterns such as PDs. The retrospective nature of the study introduces potential selection bias. Patients were not randomly selected but rather included based on the availability of data. Consequently, the study sample may not be fully representative of the broader population of individuals with BTs. For instance, in our cohort, PDs were more frequently observed in tumors located in the parietal and occipital lobes, but this may be attributable to the limited sample size or selection bias inherent in retrospective analyses. Future larger multicenter studies employing standardized EEG protocols are warranted to validate our observations.

### Clinical Relevance or Future Directions

This study delineates the prevalence and characteristics of PDs in patients with BT, with particular emphasis on their association with BTRE and SE. PDs are more prevalent in high-grade BT, especially in the parieto-occipital regions, and among patients with a prior diagnosis of BTRE. Clinically, a mean PD frequency threshold of 1.6 Hz is associated with SE in BT patients. These findings underscore the importance of individualized treatment approaches, recognizing PDs as a potential ictal pattern even at lower frequencies in this population. Despite limitations related to its retrospective design and sample size, the study highlights the necessity for prospective investigations to optimize PD management in this complex clinical context.

## Supplementary Material

npaf106_Supplementary_Data

## Data Availability

The data that support the findings of this study are available from the corresponding author upon request.
